# Creep Properties and Corrosion Behavior of TP347H Stainless Steel with Al in Molten Carbonate Salt

**DOI:** 10.3390/ma17246108

**Published:** 2024-12-13

**Authors:** Qian Meng, Lin Lai, Wan Rao, An Li, Haicun Yu, Peiqing La

**Affiliations:** 1 School of Materials Science and Engineering, Lanzhou University of Technology, Lanzhou 730050, China; hcyu@lut.edu.cn; 2 School of Materials Science and Engineering, Lanzhou Jiaotong University, Lanzhou 730070, China; lailin0325@163.com (L.L.); 13591830336@163.com (W.R.); 3 State Key Laboratory of Advanced Processing and Recycling of Non-Ferrous Metals, Lanzhou University of Technology, Lanzhou 730050, China; 4 Department of Chemical Engineering, College of Petrochemical Engineering, Lanzhou University of Technology, Lanzhou 730050, China; lian2010@lut.edu.cn

**Keywords:** TP347H stainless steel with aluminum, molten carbonate salts, corrosion resistance, creep property

## Abstract

Molten carbonate salts are a promising candidate for next-generation concentrated solar power technology owing to their excellent heat storage and heat transfer properties. This represents overcoming several problems that structural materials exhibit, including severe corrosion and high-temperature creep. Alloys with an aluminum element are alternatives in this regard as they are highly resistant to corrosive environments. In this paper, the corrosion behavior in molten carbonates (Li_2_CO_3_-Na_2_CO_3_-K_2_CO_3_) and creep properties of TP347H with different aluminum contents at 650 °C were studied. The results demonstrated that the alloy corrosion rate was reduced via Al addition. The alloy with 2.5 wt.% Al exhibited the lowest corrosion rate: ~25% lower than that without Al after 1000 h of corrosion. With increasing Al content, the inner corrosion layer of the alloys transformed from a Cr-containing oxide layer to a Cr–Al-containing composite oxide layer. The addition of Al promoted the formation of a layer of continuous and dense LiFeO_2_ product on the alloy surface during early corrosion stages, which prevented the carbonate from coming into direct contact with the substrate. After 1000 h of corrosion, the surface of the alloy is mainly composed of LiFeO_2_ and LiCrO_2_. Compared to TP347H, the added Al element enhanced the strength and elongation of TP347H at 650 °C. The TP347H containing 2 wt.% Al exhibited the best high-temperature tensile properties. When the stress was 110 MPa, the lowest steady-state creep rate of the alloy containing 2 wt.% Al was 3.61 × 10^−6^, and the true stress index was 5.791. This indicates that the creep mechanism was a dislocation climb assisted by lattice diffusion.

## 1. Introduction

Concentrated solar power (CSP) is a promising, future-oriented renewable energy technology, as it can supply dispatchable and low-cost electricity from abundant but intermittent solar energy [1,2,3]. As a crucial component of CSP plants, thermal energy storage (TES) systems reduce electricity costs, enhance annual solar-to-electricity efficiency, and considerably increase the viability of CSP technology. Mixed molten nitrate salts have recently been widely used in second-generation CSP plants as media for heat storage and transfer. However, the operating temperature of TES is limited to approximately 560 °C–570 °C due to concerns associated with nitrate salt storage media about stability and corrosion, and this operating temperature limits the thermal-to-electric conversion efficiency in CSP plants [4]. So other molten salts with higher thermal stability than molten nitrates must be used. The ternary eutectic carbonate salt 33.4 wt.% Na_2_CO_3_-34.5 wt.% K_2_CO_3_-32.1 wt.% Li_2_CO_3_ is considered a promising heat transfer and storage medium for CSP plants, as its thermo-physical properties are comparable to those of molten nitrate salt and it offers a wide range of operating temperatures [5,6].

Although molten carbonates satisfy the thermal–physical requirements of a heat transfer and storage medium, elevated temperatures and the changes that occur in molten salts can exacerbate the corrosion of storage materials. Stainless steels and Ni-based alloys are the primary materials used for molten salt storage in TES within CSP plants. Investigating the corrosion performance of common storage materials in carbonate salts is still essential to CSP applications [7]. The stability of the stainless steels and Ni-based alloys that come into contact with heat transfer fluids is crucial for the longevity of CSP systems [8]. Most researchers have focused on the corrosion behavior of stainless steel in molten carbonate salts when studying salt storage materials for CSP plants. Sah et al. [9] compared the corrosion behavior of various austenitic stainless steels (AFAs) in molten carbonate at 650 °C. Their study found that Cr substantially improved the passivation of the alloy surface. Additionally, high levels of both Cr and Ni greatly enhanced an alloy’s resistance to corrosion. Takeuchi et al. [10] investigated the corrosion behavior of binary Fe-Cr alloys in various Na_2_CO_3_–K_2_CO_3_ compositions. They found that the oxidation products formed by Cr enhanced the alloy’s resistance to metal ion diffusion, and thus the alloy showed high corrosion resistance; however, the Cr and Fe in an alloy both dissolve in molten carbonate. Additionally, de Miguel et al. [11] investigated the corrosion behavior of HR3C in carbonate at 700 °C. They found that Cr reacted with the carbonate during the corrosion process to form the highly soluble compound K_2_CrO_4_ and that the content of Cr in the carbonate considerably increased after corrosion. Although Cr can serve as a protective element in stainless steel, excessive amounts of Cr may lead to the formation of soluble chromates in molten carbonate. This results in the loss of Cr and limits the involved protective effect on the substrate [12]. Compared with stainless steel, Ni-based alloys show better corrosion resistance in molten salts [13,14,15], and those with a higher Cr content are more prone to forming protective Ni-based spinel in molten carbonate environments. This spinel formation enhances corrosion resistance, as it acts as a barrier that protects the underlying metal from aggressive species in a molten medium [16,17]. In nitrate-based CSP, TP347H stainless steel is commonly used as a salt storage material due to its excellent high-temperature mechanical properties and corrosion resistance [18]. However, Prieto et al. [19] has revealed that at 710 °C in a ternary eutectic carbonate environment, 347H stainless steel experiences significant corrosion, with 180 to 210 μm/year. Various anti-corrosion methods that target both the molten salts and the alloys themselves have been proposed to reduce the corrosion rate of stainless steel in molten salts [20,21,22,23,24,25]. The main anti-corrosion methods include the formation of protective coatings on the alloy surface, the incorporation of alloying elements, and the addition of corrosion inhibitors to the molten salts. Unfortunately, challenges arise that are related to surface modification and the use of corrosion inhibitors, including problems like coating delamination, cracking, and the difficulty of controlling the amount of inhibitor applied. However, the incorporation of alloying elements can create a dense and stable protective film on the stainless-steel surface, thereby improving its corrosion resistance. Furthermore, trace elements are added to enhance mechanical strength and corrosion/oxidation resistance at high temperatures.

Current research on the addition of alloying elements focuses on Si, Al, Cu, Mo, and rare-earth elements. Among these elements, Al has garnered considerable attention for its ability to form an Al_2_O_3_ oxide layer on the surface when it is added to stainless steel; this provides high oxidation and corrosion resistance [26,27]. Researchers have discovered that when the Al content in alumina-forming austenitic (AFA) steels was adjusted, a dense Al_2_O_3_ oxide layer can form on the surface of the AFAs. This layer offers greater high-temperature stability and corrosion resistance than a Cr_2_O_3_ oxide layer. Meanwhile, aluminum can promote the formation of B2-NiAl precipitate, thus significantly enhancing the creep resistance of alloys [28,29,30,31,32]. Hamdy et al. [33] studied the corrosion behavior of AFAs in molten carbonate and found that their corrosion rate was substantially lower than that of other types of stainless steel. Salt storage materials that are used in CSP plants must exhibit both corrosion resistance and high-temperature creep resistance on exposure to a high-temperature molten salt environment. Existing research on alumina-forming austenitic (AFA) steels primarily emphasizes corrosion resistance. However, few studies have investigated the synergistic effects of Al and Cr on corrosion in molten carbonates and the involved creep properties at high temperatures.

Herein, TP347H plates with different levels of Al content (0, 1.5, 2, and 2.5 wt.%) were designed and prepared based on the alloy composition of TP347H stainless steel for salt storage material. The corrosion behavior of the alloys was systematically investigated in the eutectic LiNaK molten carbonate salt, and the high-temperature tensile and creep properties of the alloys were compared at 650 °C. This research aims to provide theoretical support for the design of materials that are suitable for the storage and transportation of heat transfer media in CSP technology.

## 2. Materials and Methods

### 2.1. Material Preparation

Based on the composition of TP347H, various alloy compositions were designed by incorporating Al to reduce the Cr content. Table 1 shows the compositions of the alloys. The raw materials, which were prepared according to each designed composition, were melted using a vacuum suspension melting furnace to obtain 4.5 kg ingots. From each ingot, a 15 mm thick block sample was cut and rolled into a 6 mm thick sheet at a temperature of 1200 °C. After it was rolled, the sheet was heated to 1050 °C and kept at this temperature for 30 min to complete the solid solution treatment, which was followed by water cooling.

### 2.2. Corrosion Test

**Figure 1:**
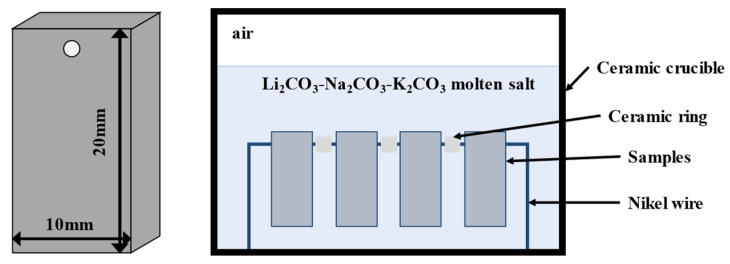
Specimen arrangement for the static corrosion test.

The corrosion test was conducted in an atmospheric environment at 650 °C. Corrosion specimens sized 20 × 10 × 6 mm^3^ were prepared using electrical discharge machining, grinded and polished, and then cleaned ultrasonically in alcohol to remove any residual impurities. The weight of the specimens was measured using an analytical balance with an accuracy of 0.0001 g. The specimens were suspended as shown in Figure 1. The mixed ternary eutectic carbonate Li_2_CO_3_-Na_2_CO_3_-K_2_CO_3_ (32.1–33.4–34.5 wt.%) was poured into a crucible, and the specimens were completely submerged. The crucible was then covered, placed in a muffle furnace, and heated to 650 °C for 100, 200, 300, 400, 500, and 1000 h, respectively. We removed the crucible and cooled to room temperature at each time point.

After the desired corrosion time had elapsed, the specimens were removed from the molten salt, air-cooled to room temperature, and cleaned with boiling deionized water and a 10% H_2_SO_4_ solution to remove residual salts from their surface. The specimens were then cleaned with alcohol, dried, and weighed to record any changes in their weight. The mass gain over time of the samples was calculated by Equation (1), and the corrosion rate was calculated by Equation (2):

(1) $$\frac{∆m}{S_{0}}=\frac{m_{f}-m_{i}}{S_{0}},$$

where m_i_ is the initial mass of the sample, m_f_ is the mass of the sample at the desired time, and S_0_ is the initial surface area of the sample.

(2) $$\mathrm{CR}\left(\mu m/\mathrm{year}\right)=\frac{K\times W}{A\times t\times \rho },$$

where K is a constant value of 8.76 × 107 (μm·h·cm^−1^·year^−1^); W is the mass lost by the sample (g); A is the surface area of the sample (cm^2^); t is corrosion time (h); and ρ is the sample density (g/cm^3^).

### 2.3. Creep Property Test

This study conducted high-temperature instantaneous tensile experiments according to the standard procedure GB/T 228.2-2015 [34]. A CMT 5305 300-kN high-temperature tensile testing machine was used to test the high-temperature instantaneous tensile properties of the alloys containing different Al contents at 650 °C. According to the standard procedure GB/T 2039-2012 [35], the creep property of TP347H with 2 wt.% Al was tested with a high-temperature persistent creep testing machine (GWT 2105 100 kN, San-si-zong-heng, Shenzhen, China) at 650 °C.

### 2.4. Microstructure Characterization

The microstructure of the alloy with different Al contents was characterized using an option microscope (XZJ-L2030A, A-er-ma in Shenzhen, China) and X-ray diffractometer (XRD-7000, Shimadzu, Kyoto, Japan). A field-emission scanning electron microscope (Gemini 500, Zeiss, Tokyo, Japan) using an energy dispersive X-ray spectrometer (EDS) was used to observe the fracture morphology of high-temperature creep specimens, as well as the surface and cross-section morphology of the corroded specimens.

## 3. Results

### 3.1. Microstructure and Morphology

**Figure 2:**
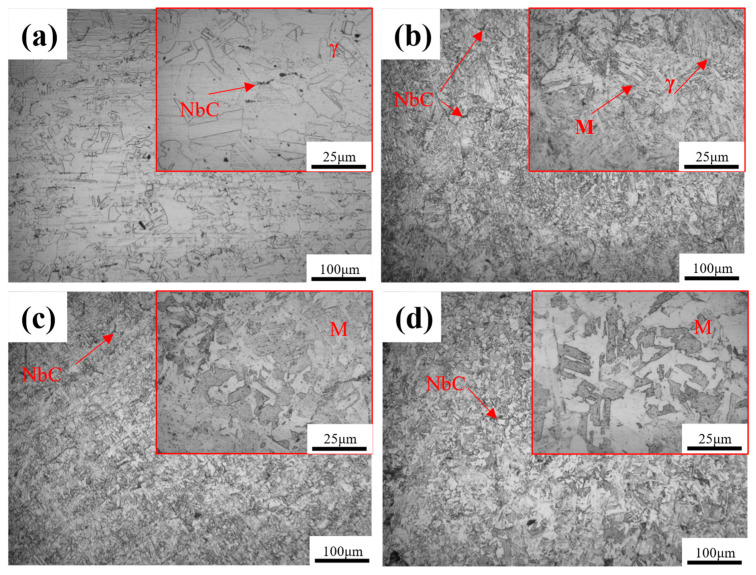
Metallographic structure of the alloy with different Al contents: (**a**) 0 wt.% Al; (**b**) 1.5 wt.% Al; (**c**) 2 wt.% Al; (**d**) 2.5 wt.% Al.

**Figure 3:**
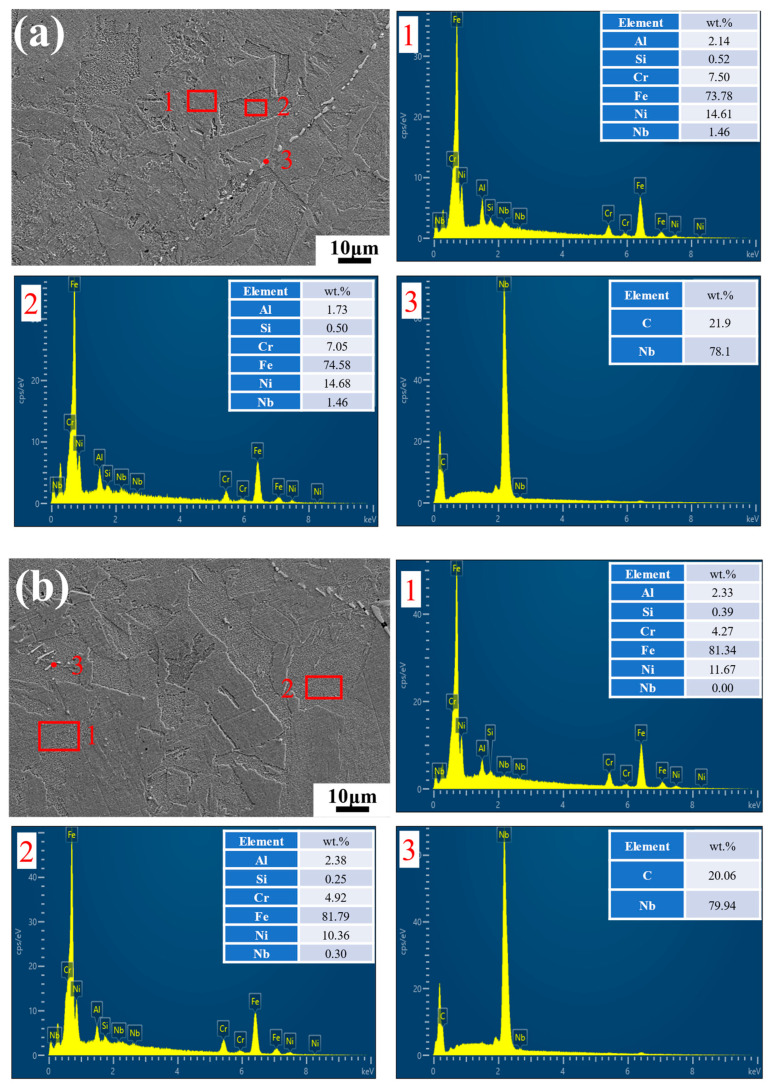
SEM images and EDS results for the alloys with different Al contents: (**a**) 2 wt.% Al; (**b**) 2.5 wt.% Al.

Figure 2 shows the metallographic structure of the alloy specimens with different Al contents after solid solution treatment. The microstructure in the alloy without Al was single-phase austenite (Figure 2a). With an increase in Al content, the microstructures were transformed from a single-phase austenitic structure to a dual-phase structure comprising martensite and austenite (Figure 2b–d). Martensite exhibited a slatted and blocky shape in the alloy specimens with 2 and 2.5 wt.% Al. EDS was conducted in different regions, and it revealed no notable differences in the elemental composition between various locations within the matrix (Figure 3). A discontinuous, point-like second phase was observed within the matrix and identified as NbC through EDS analysis (Figure 3(a3,b3)). Furthermore, no precipitated phases containing Al were detected in the matrix, which indicated that the Al was entirely dissolved within it.

### 3.2. Corrosion Resistance Analysis

**Figure 4:**
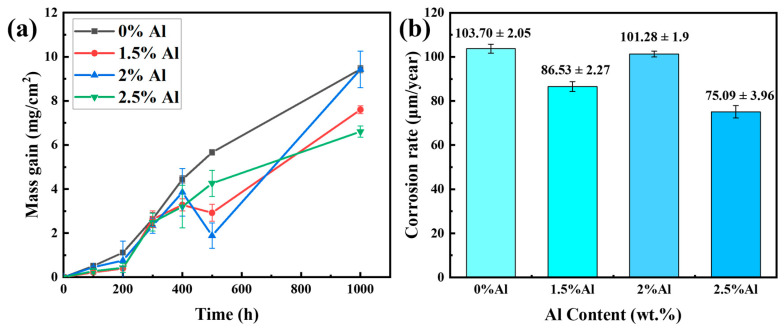
(**a**) Corrosion weight gain curve of the alloys with different Al contents; (**b**) corrosion rate of the alloys with different Al contents after 1000 h of corrosion.

**Figure 5:**
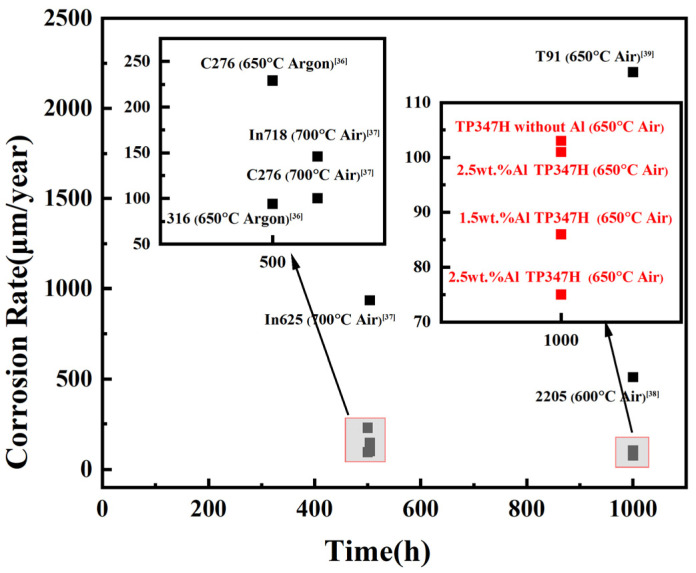
Corrosion rate of different alloys in carbonate [36,37,38,39].

The curves depicting the weight gain of TP347H steel with different Al contents during corrosion in molten carbonate are shown in Figure 4a. For the sample of the alloy without Al, the kinetic curve exhibits linearity throughout the entire corrosion process, which indicates that the corrosion layer on the sample’s surface grows uniformly. After 1000 h of exposure, the sample exhibits severe corrosion, with a weight gain of approximately 9.44 mg/cm^2^. However, for the samples of the alloy containing Al, the weight gain is slightly lower than that of the sample without Al, particularly for the alloy with 2.5 wt.% Al. The weight gain curve for this sample resembles a parabola, which indicates that a protective layer of corrosion product formed on its surface during the corrosion process. This protective layer effectively slows down the rate of the growth of corrosion products in subsequent exposure periods and enhances the overall corrosion resistance of the material. The corrosion rate for the alloys containing different Al contents in molten carbonate after 1000 h of exposure is shown in Figure 4b. Notably, the corrosion rate of the alloy with 2.5 wt.% Al is the lowest at 75.09 ± 3.96 μm/year, which is approximately 25% lower than that of the alloy without Al. This indicates that adding Al substantially enhances the corrosion resistance of the alloy in molten carbonate environments. As shown in Figure 5, other relevant research results show that the corrosion rate in carbonate of the alloy with 2.5 wt.% Al is lower than that of several commonly used alloys [36,37,38,39].

**Figure 6:**
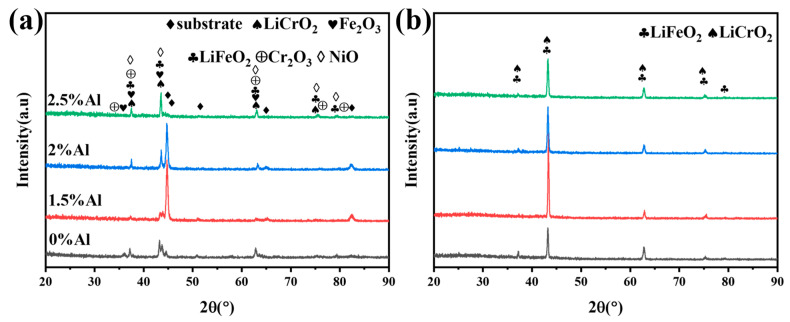
XRD pattern of the alloy with different Al contents after different corrosion times: (**a**) 200 h; (**b**) 1000 h.

Figure 6 shows the X-ray diffraction (XRD) patterns of TP347H steel samples containing different Al contents after 200 and 1000 h of corrosion. As shown in Figure 6a, it is evident that after 200 h of corrosion, peaks for LiFeO_2_, LiCrO_2_, Fe_2_O_3_, Cr_2_O_3_, NiO, and matrix appear on the surfaces of all four steel groups. With an increase in the Al content in the alloy, the peak of LiFeO_2_ and LiCrO_2_ becomes more obvious, while the matrix peak gradually weakens. The surface of the alloy with 2.5 wt.% Al contains hardly any matrix peak. It can be observed that the corrosion products of the four steel samples are mainly composed of LiFeO_2_ and LiCrO_2_ after 1000 h, as shown in Figure 6b.

**Figure 7:**
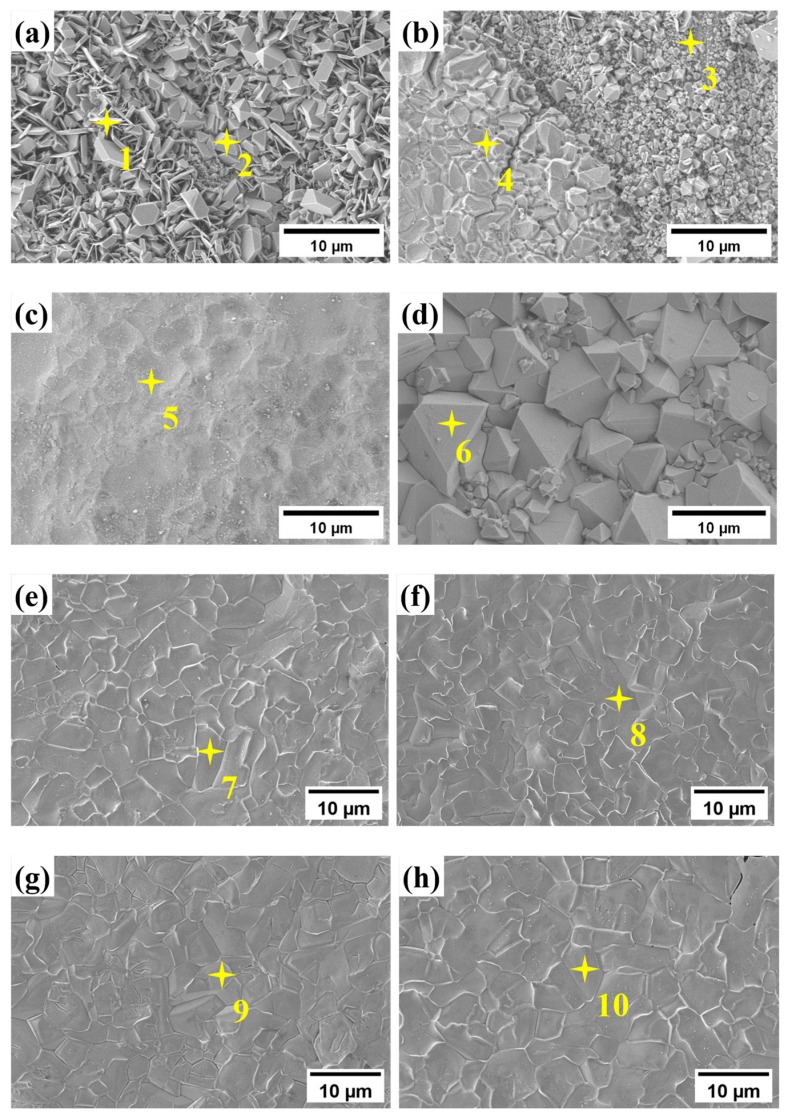
SEM image and surface of TP347H steel with different Al contents after corrosion: (**a**) 0 wt.% Al-200 h; (**b**) 1.5 wt.% Al-200 h; (**c**) 2 wt.% Al-200 h; (**d**) 2.5 wt.% Al-200 h; (**e**) 0 wt.% Al-1000 h; (**f**) 1.5 wt.% Al-1000 h; (**g**) 2 wt.% Al-1000 h; (**h**) 2.5 wt.% Al-1000 h.

Figure 7 shows the surface SEM morphology of the four samples after 200 and 1000 h of corrosion, and the corresponding EDS analysis results are presented in Table 2. After 200 h of corrosion, the surface of the four groups of samples shows different microstructures. The corrosion products on the surface of the alloy containing 0 wt.% Al comprise spinel-shaped and flaky-shaped corrosion materials (Figure 7a). The EDS analysis indicates that the flake-shaped products are mainly composed of Fe, Cr, and O, while the spinel-shaped products are composed of Fe and O. Combining the XRD results, it can be concluded that the flake-shaped products are Cr_2_O_3_ and Fe_2_O_3_ phases, while the spinel-shaped products are LiFeO_2_. Since the atomic number of Li is smaller, EDS cannot detect it. The corrosion products in different Al content alloys are mainly in the form of spinel-shaped products, and as the Al content increases, the size of the spinel-shaped products increases and connects to form a dense corrosion layer, gradually covering the entire surface (Figure 7b–d). Both EDS and XRD analyses reveal that this corrosion layer primarily comprises LiFeO_2_ and LiCrO_2_, which indicates that LiFeO_2_ and LiCrO_2_ form more rapidly in steels with an elevated aluminum content. Figure 7e–h show that a continuous and dense LiFeO_2_ and LiCrO_2_ corrosion layer is formed on the surface of all samples after 1000 h of corrosion.

**Figure 8:**
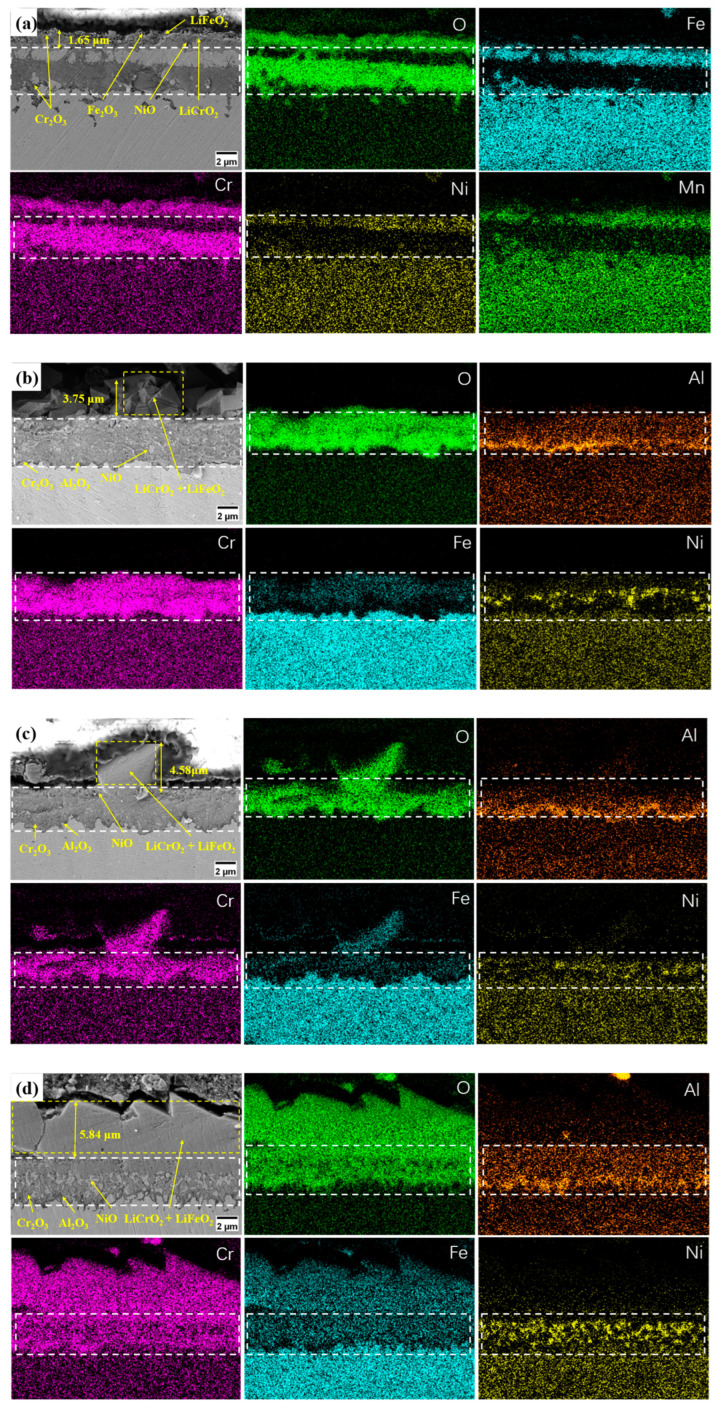
SEM images of and EDS results for the corrosion cross-section in the alloys with different Al contents after 200 h of corrosion: (**a**) 0 wt.% Al; (**b**) 1.5 wt.% Al; (**c**) 2 wt.% Al; (**d**) 2.5 wt.% Al.

**Figure 9:**
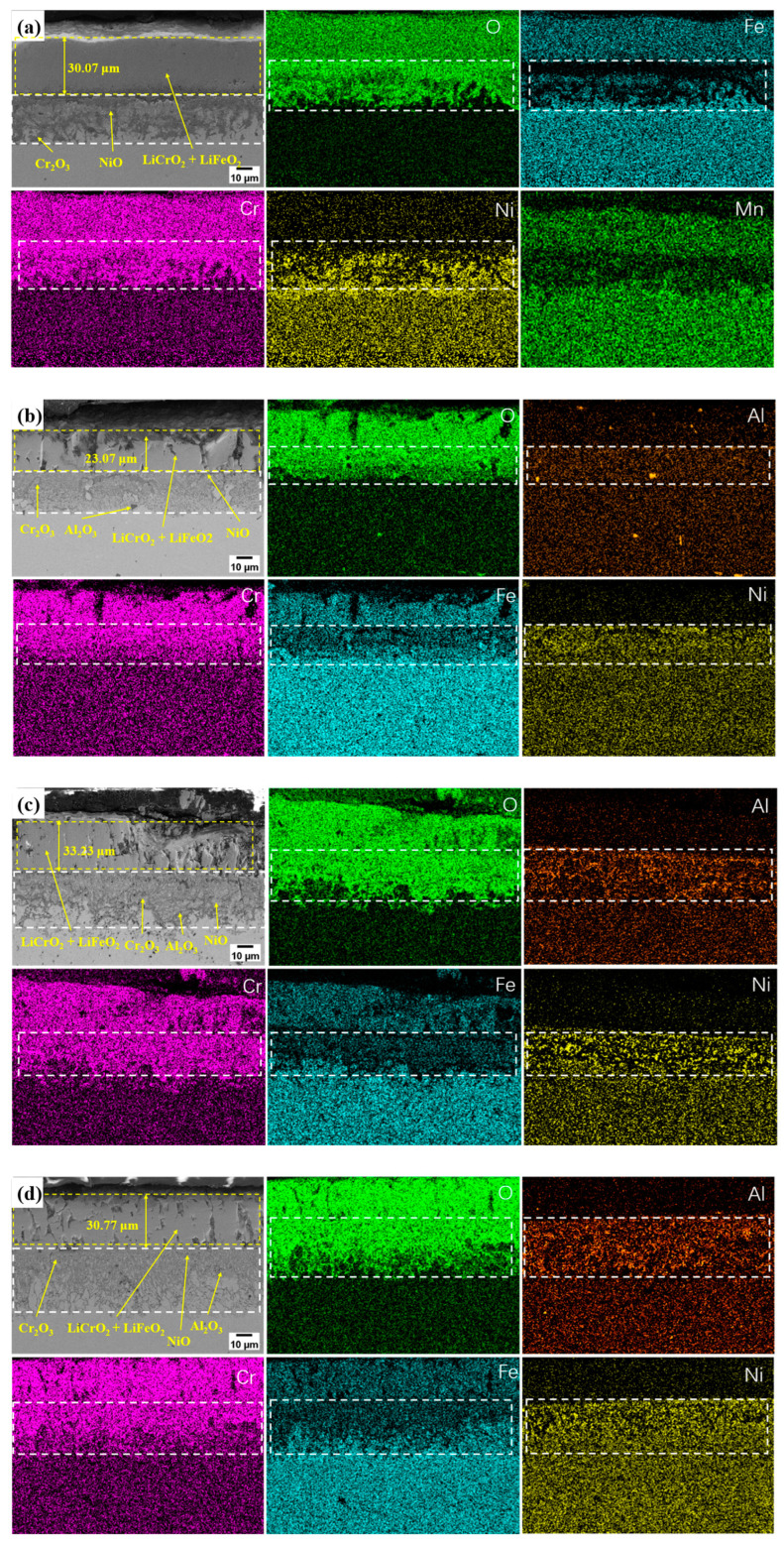
SEM images of and EDS results for the corrosion cross-section in the alloys with different Al contents after 1000 h of corrosion: (**a**) 0 wt.% Al; (**b**) 1.5 wt.% Al; (**c**) 2 wt.% Al; (**d**) 2.5 wt.% Al.

Figure 8 and Figure 9 show cross-sectional SEM-EDS images of the TP347H samples containing different Al contents after 200 and 1000 h of corrosion, respectively. After 200 h of corrosion, it can be observed that the surface of the alloy with 0 wt.% Al forms an outer corrosion layer with a thickness of about 1.65 microns, mainly composed of LiFeO_2_, LiCrO_2_, Cr_2_O_3_, Fe_2_O_3_, and NiO (Figure 8a). The inner corrosion layer is mainly enriched with Cr and O elements, which indicates that the layer is mainly composed of Cr_2_O_3_ (Figure 8a). Additionally, a large number of Fe elements diffuse from the inner corrosion layer to the outer corrosion layer, as can be observed in Figure 8a. The alloy with 1.5-, 2-, and 2.5 wt.% Al, which formed an outer corrosion layer comprising LiFeO_2_ and LiCrO_2_. With an increase in Al content in this alloy, the thickness of the outer corrosion layer also increased from about 3.75 μm to over 5 μm. An enrichment of Cr, Al, Ni, and O was observed at the inner corrosion layer, indicating that the inner corrosion layer is primarily comprised of Cr_2_O_3_, Al_2_O_3_, and NiO, and the thickness of the aluminum oxide layer in the inner corrosion layer increased (Figure 8b–d). Compared to the alloy without Al, NiO exists in the inner corrosion layer rather than the outer corrosion layer of the alloy containing aluminum. After 1000 h of corrosion, the outer corrosion layer of the four groups of samples comprised continuous and dense LiFeO_2_ and LiCrO_2_ (Figure 9a–d). The degree of the enrichment of Al in the inner corrosion layer increased with the increase in the Al content, and the area of Fe elements diffused in the inner corrosion layer decreased gradually, which indicated that the Al_2_O_3_, Cr_2_O_3_, and NiO composite layer had a certain inhibitory effect on the outward diffusion of the elements in the alloy.

### 3.3. Creep Property Analysis

The high-temperature instantaneous tensile properties of TP347H samples with different Al contents are shown in Table 3. The alloy containing 2 wt.% Al exhibits the highest tensile properties. Its elongation is increased by 24.5% relative to the alloy without Al. These results indicate that the added Al element enhanced the strength and elongation of TP347H at 650 °C.

**Figure 10:**
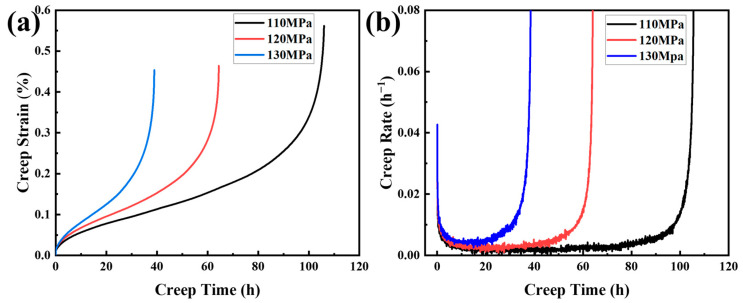
Creep strain curves and creep rate curves of the alloy with 2 wt.% Al under different stress at 650 °C: (**a**) creep time–strain curve; (**b**) creep time–creep rate curve.

The alloy containing 2 wt.% Al content, which was noted for its superior tensile properties in high-temperature instantaneous tensile tests, was subjected to high-temperature creep experiments at 650 °C under different stresses. Figure 10 shows the creep strain and creep rate curves of the alloy under different values of constant stress. As shown in Figure 10a, the alloy exhibits three stages that are typical of creep under different constant stress conditions, i.e., the initial stage when the creep rate decreases over time, the steady-state stage when the creep rate is almost constant, and the third stage when the creep rate increases sharply to fracture. The total creep rupture life of the specimens decreases with an increase in the applied stress. Figure 10b reveals that the steady-state creep rate increases with a higher applied stress. Table 4 summarizes the fracture times and minimum creep rates of the alloy under different stress conditions. When the stress is 110 MPa, the creep rupture life of the alloy is 106 h, and the minimum creep rate is 3.61 × 10^−6^ h^−1^.

**Figure 11:**
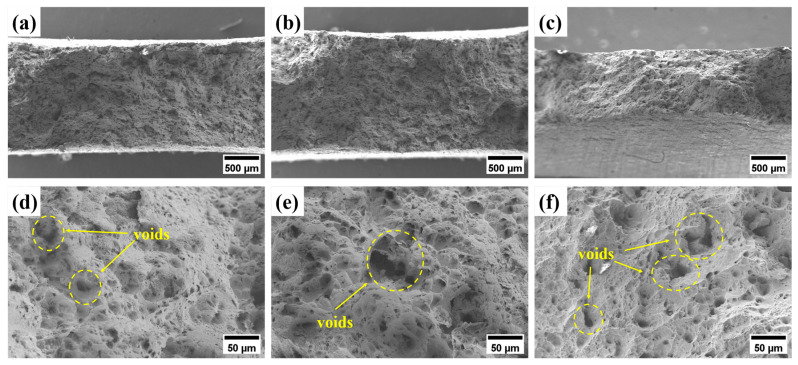
SEM images of the creep fracture morphology of the alloy containing 2 wt.% Al under different stresses: (**a**,**d**) 110 MPa; (**b**,**e**) 120 MPa; (**c**,**f**) 130 MPa.

Figure 11 shows the SEM images for the creep fracture morphology of the alloy containing 2 wt.% Al at 650 °C. Notably, it exhibits a ductile fracture mode at different stress values, and there are numerous unevenly distributed holes at the fracture. With an increase in applied stress, the number, size, and depth of the holes at the fracture decrease to different degrees, which is attributed to the fact that the main precipitation phases in aluminum-containing stainless steels included MC(NbC), M_23_C_6_(Cr_23_C_6_), B2-(Ni, Fe)Al, and Laves(Fe_2_Nb) phases [26,28]. At higher stresses, dislocations near the precipitated phases tend to accumulate, and the stress concentration is severe, which induces crack initiation and promotes crack extension, at which time fracture occurs before the holes have time to expand, thus resulting in poor creep properties.

## 4. Discussion

### 4.1. Corrosion Resistance

**Figure 12:**
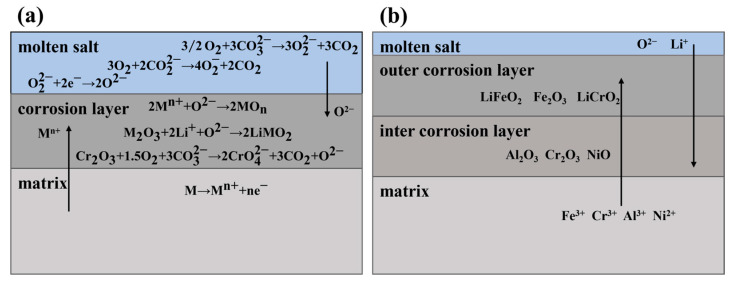
Sketch of formation process for corrosion layer: (**a**) the initial stage of corrosion; (**b**) stable corrosion stage.

Figure 12 presents a schematic diagram of the process of alloy corrosion in carbonate. During the corrosion process, the molten carbonate is in direct contact with air. The carbonate ions in the molten carbonate salts react with the dissolved oxygen, which results in the formation of $O_{2}^{2-}$ and $O_{2}^{-}$, as described by the reaction in Equations (3) and (4) [40].

(3) $$3/2O_{2}+3{\mathrm{CO}}_{3}^{2-}\to 3O_{2}^{2-}+3{\mathrm{CO}}_{2}$$

(4) $$3O_{2}+2{\mathrm{CO}}_{2}^{2-}\to 4O_{2}^{-}+2{\mathrm{CO}}_{2}.$$

During the initial stage of corrosion, an electrochemical reaction occurs between the alloy and the molten carbonate salts. Metal cations and electrons from the surface of the alloy are ionized. The $O_{2}^{2-}$ and $O_{2}^{-}$ in the molten carbonate salts obtain the electron-generated $O^{2-}$, which combines with the metal cations on the alloy surface to form an oxide, as shown in the reaction in Equations (5)–(7) [41].

(5) $$M\to M^{n+}+{\mathrm{ne}}^{-}$$

(6) $$O_{2}^{2-}+2e^{-}\to {2O}^{2-}$$

(7) $${2M}^{n+}+O^{2-}\to 2MO_{n}.$$

Here, M means Fe, Cr, Ni, and Al. During the corrosion process, the oxide that forms on the surface of the sample is in direct contact with the carbonate and subsequently reacts with the Li⁺ in the carbonate. Additionally, some chromium oxide reacts with ${\mathrm{CO}}_{3}^{2-}$ in the carbonate to form soluble chromate, as shown in the reaction in Equations (8) and (9) [42,43].

(8) $$M_{2}O_{3}+2{\mathrm{Li}}^{+}+O^{2-}\to 2{\mathrm{LiMO}}_{2}$$

(9) $${\mathrm{Cr}}_{2}O_{3}+1.5O_{2}+3{\mathrm{CO}}_{3}^{2-}\to \;2{\mathrm{CrO}}_{4}^{2-}+3{\mathrm{CO}}_{2}+O^{2-}.$$

During the process of carbonate corrosion, TP347H containing different Al contents underwent successive oxidation and lithium processes. During the initial corrosion stage, oxidation reactions occurred on the specimen surface to form oxides such as Fe_2_O_3_, Cr_2_O_3_, and Al_2_O_3_. These oxides then reacted with the carbonates that were in direct contact to form spinel and flaky corrosion products, including LiFeO_2_ and LiCrO_2_. As the corrosion continued, these products grew rapidly and covered the specimen surface. Among the products, Cr_2_O_3_ reacted with the carbonate, a portion of it formed soluble chromate dissolved in the carbonate, the other part formed LiCrO_2_ and remains on the specimen surface with LiFeO_2_, whereas the spinel-like LiFeO_2_ gradually grew and fused into a block, eventually covering the entire surface of the sample. Because LiFeO_2_ is almost insoluble in the molten carbonate, the dense LiFeO_2_ prevents the molten salt from further corroding the matrix, thereby serving as a protective outer corrosion layer on the specimen surface.

In alloys containing Al, the oxygen absorption capacity of the elements follows the order: Al > Si > Cr > Fe > Ni [44]. When Al is oxidized to form Al_2_O_3_, Cr and Fe are also oxidized to form their corresponding oxides. However, because the content of Cr in the alloy with added Al element is less than 16 wt.%, which is lower than the critical content required for the formation of a protective Cr oxide layer [45], the capacity for stable and continuous Cr oxide formation on the specimen surface decreases. In this case, the outward diffusion of matrix elements is not effectively hindered. Moreover, the low Cr content weakens the “third element effect” that is provided by Cr when Al_2_O_3_ is formed. As a result, the form of oxidation within the formed Al_2_O_3_ remains in the matrix, and the formation of the Al_2_O_3_ layer undergoes a transition from unstable to stable. During the initial stages of corrosion, the Cr_2_O_3_, NiO, and Al_2_O_3_ layers formed on the alloy surface cannot effectively prevent the outward diffusion of internal matrix elements. This permits a considerable amount of iron to oxidize into Fe_2_O_3_ without the establishment of a stable Cr_2_O_3_-Al_2_O_3_ composite oxide layer. Additionally, Fe_2_O_3_ reacts with Li_2_CO_3_ in the molten carbonate salts to form LiFeO_2_ and LiCrO_2_. Thus, a protective LiFeO_2_ outer corrosion layer is formed faster on the surface of the alloys containing a higher Al content and a lower Cr content. For instance, after 200 h of corrosion, the surface of the alloy with 2.5 wt.% Al is completely covered by LiFeO_2_ and LiCrO_2_.

During the stable corrosion stage, oxygen continues to diffuse into the matrix, which further oxidizes Cr and Al to Cr_2_O_3_ and Al_2_O_3_, respectively. The oxidation regions within the inner corrosion layer gradually grow and connect with each other to form a Cr_2_O_3_-Al_2_O_3_ composite layer. When this composite layer becomes stable and dense, it can further hinder the outward diffusion of matrix elements better than a single Cr_2_O_3_ oxide layer; this slows down the growth rate of the outer corrosion layer of the alloy, and thus the alloy shows a lower corrosion rate. Consequently, the TP347H alloy with the addition of Al demonstrates improved corrosion resistance.

### 4.2. Creep Properties

To further understand the relation between the minimum creep rate of the material and the stress, the temperature-compensated power law creep equation shown in Equation (10) is used for analysis [46]:

(10) $${\dot{\varepsilon }}_{s}=A\sigma^{n}\exp(-\frac{Q}{\mathrm{RT}}),$$

where ${\dot{\varepsilon }}_{s}$ is the minimum creep rate, A is a material constant, σ is the applied stress, n is the stress exponent, Q is the activation energy required for creep, R is the universal gas constant, and T is the absolute temperature (K). Under constant temperature conditions, the steady-state creep rate ${\dot{\varepsilon }}_{s}$ used in this experiment has a power law relation with the applied stress σ, as shown in Equation (11):

(11) $${\dot{\varepsilon }}_{s}=A\sigma^{n}.$$

**Figure 13:**
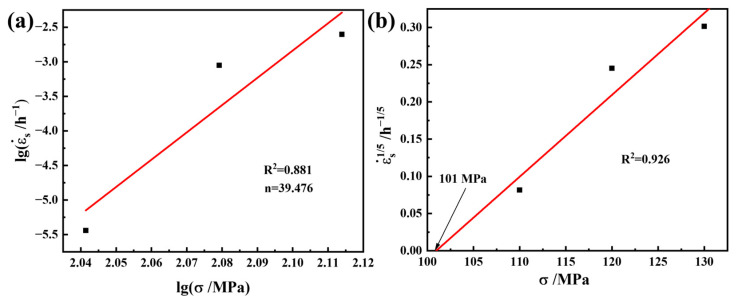
(**a**) Logarithmic curve of the minimum creep rate versus creep stress of the alloy with 2 wt.% Al. (**b**) Variation in minimum creep rate to the power of 1/5 versus creep stress of the alloy with 2 wt.% Al.

**Figure 14:**
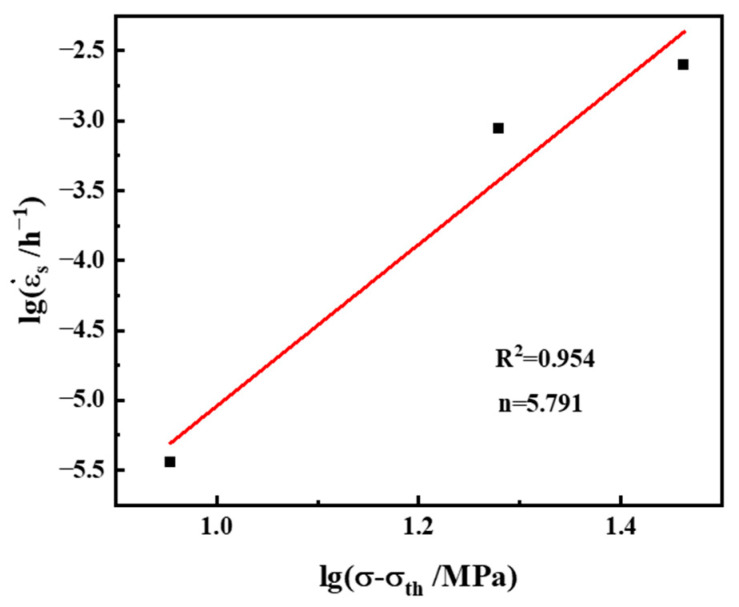
Logarithmic curve of minimum creep rate and true stress of the alloy with 2 wt.% Al.

When Equation (10) is expressed logarithmically, lg${\dot{\varepsilon }}_{s}$ and lgσ exhibit a linear relation; moreover, the stress index n can be obtained after the linear fitting of lg${\dot{\varepsilon }}_{s}$ − lgσ (Figure 13a). The creep stress index n of the alloy containing 2 wt.% Al is 39.476. During the creep process, when the stress exponent is greater than 5, the second phase particles can significantly affect the movement of the dislocations. Dislocations need additional stress to cross the second phase particles; this additional stress is called the threshold stress σ_th_. The existence of a stress threshold causes the apparent stress index of the material to be higher than the actual stress index, and thus its existence cannot effectively reflect the creep mechanism of the material. Therefore, the threshold stress of the material must be calculated to obtain the real stress index. The creep threshold stress is obtained through linear fitting of the minimum creep rate to the power of 1/n (n = 3, 5, and 8) and the stress. The n value with the best linear correlation is selected and extrapolated by linear regression. Figure 13b shows the linear fitting curve for the minimum creep rate to the power of 1/5. The threshold stress σ_th_ of the alloy with 2 wt.% Al at 650 °C is 101 MPa, from which the linear relation between the minimum creep rate ${\dot{\varepsilon }}_{.}$ and the true stress (σ − σ_th_) can be obtained (Figure 14), and the true stress index of the material is determined to be 5.791. These results indicate that the creep mechanism is a dislocation climb assisted by lattice diffusion [47].

## 5. Conclusions

Herein, TP347H containing different Al contents was prepared, and its corrosion behavior in molten carbonate salts and creep properties at 650 °C was tested. The following conclusions were drawn:

1. The matrix microstructure of the TP347H with Al changes from austenite to austenite and martensite. The Al element dissolved in the matrix. The corrosion rate of the TP347H with 2.5 wt.% Al content was 75.09 ± 3.96 μm/year for 1000 h, which was approximately 25% lower than the alloy without the Al element. The addition of the Al element is beneficial for the formation of a continuous and dense Cr_2_O_3_, Al_2_O_3_, and NiO composite layer in the inner corrosion layer of the alloys. The composite oxide layer effectively protected the matrix, which improved the corrosion performance of the alloy in the molten salt.
2. The addition of the Al element promoted the formation of a dense LiFeO_2_ layer on the surface of the alloy in the early stage of corrosion. Al is a strong oxygen-absorbing element, and it promoted the formation of iron and chromium oxides on the specimen surface during high-temperature corrosion. The reaction of iron oxides with molten salt formed LiFeO_2_. This reduced the formation of chromate, which is highly soluble in carbonate, and thus reduced the dissolution of the matrix elements into the molten salt.
3. At 650 °C, the fracture characteristic of the alloys was ductile fracture. The lower the creep stress, the longer the fracture time and the lower the steady-state creep rate for alloys containing 2 wt.% Al content. When the creep stress was 110 MPa, the lowest steady-state creep rate was 3.61 × 10^−6^. The true stress index was 5.791, and the deformation mechanism in the creep process was a lattice diffusion-assisted edge-type dislocation shift control mechanism.

## Figures and Tables

**Table 1:** Composition of the alloy with different Al contents (wt.%).

Sample	Al	C	Cr	Ni	Mn	Si	Nb	Fe
0 wt.% Al	0	0.07	18	11.5	1.18	0.5	0.7	Bal.
1.5 wt.% Al	1.5	0.07	9.75	11.5	1.18	0.5	0.7	Bal.
2 wt.% Al	2	0.07	7	11.5	1.18	0.5	0.7	Bal.
2.5 wt.% Al	2.5	0.07	4.25	11.5	1.18	0.5	0.7	Bal.

**Table 2:** EDS element analysis at different locations in the sample in Figure 7.

EDS (wt.%)	Region									
	1	2	3	4	5	6	7	8	9	10
O	35.0	33.7	20.6	26.9	28.0	31.4	32.0	39	34.3	32.8
Al	-	-	10.5	0.7	0.4	0.2	-	0.4	0.2	0.2
Cr	12.3	2.7	9.1	2.4	0.7	0.1	0.1	0.0	0	0.1
Mn	1.4	2.5	1.1	1.3	0.7	1.1	3.1	1.9	1.9	0.9
Fe	50.3	60.3	52.4	68.0	70.0	67.0	64.3	58.4	62.5	65.3
Ni	0.0	0.4	6.5	0.8	0.0	0.0	0.2	0.2	0.7	0.2

**Table 3:** High-temperature instantaneous tensile properties of TP347H samples containing different Al contents.

Sample	YS (MPa)	UTS (MPa)	EL (%)
0 wt.% Al	164	266	31
1.5 wt.% Al	219	277	32.5
2 wt.% Al	256	278	55.5
2.5 wt.% Al	234	262	46

**Table 4:** Creep rupture time and minimum creep rate of the alloy.

Sample	Stress (MPa)	Creep Time (h)	Minimum Creep Rate (h^−1^)
2 wt.% Al	110	106	3.61 × 10^−6^
	120	64.3	8.86 × 10^−4^
	130	38.9	2.49 × 10^−3^

## Data Availability

The original contributions presented in the study are included in the article; further inquiries can be directed to the corresponding authors.
